# Supplementary material for: MetaRibo-Seq measures translation in microbiomes
Source: Nat Commun. 2020 Jun 29;11:3268. doi: 10.1038/s41467-020-17081-z (PMC7324362; doi:10.1038/s41467-020-17081-z)
Supplement: Supplementary file 10 — Supplementary Data 7 [file 41467_2020_17081_MOESM10_ESM.zip › File2/Confidence_High_Taxonomy/14131_out.krona.html]

Javascript must be enabled to view this page.

members
magnitude
magnitudeUnassigned
count
unassigned
taxon
rank

14131\_out

3

3
2
superkingdom

phylum
3
976

class
3
200643

order
3
171549

family
1
815

genus
1
816

species
1

SRS049446\_contig\_number\_1806
820

171551
1
family

genus
1
836

997829
1

SRS014935\_contig\_number\_contig-100\_29763.64963
species

1
171552
family

genus
838
1

60133
1

SRS143062\_contig\_number\_14029
species
